# Plasma cell-free RNA PD-L1 or tissue PD-L1 protein expression and outcomes with first-line immunotherapy in metastatic non-small cell lung cancer

**DOI:** 10.1016/j.jlb.2023.100130

**Published:** 2023-12-04

**Authors:** Paul R. Walker, Sriraksha Jayananda, Melisa Pasli, Mahvish Muzaffar

**Affiliations:** a Division of Hematology/Oncology, Brody School of Medicine at East Carolina University, 600 Moye Blvd, Greenville, NC, 27834, USA; b Circulogene Theranostics, 3125 Independence Drive, Birmingham, AL, 35209, USA; c Brody School of Medicine at East Carolina University, 600 Moye Blvd, Greenville, NC, 27834, USA

**Keywords:** Liquid biopsy, PD-L1, Plasma cfRNA, Immunotherapy, NSCLC

## Abstract

**Background:**

Tissue programmed death ligand-1 (PD-L1) protein expression is associated with immune checkpoint inhibitor (ICI) treatment benefit in metastatic non-small cell lung cancer (NSCLC). However, tissue PD-L1 protein testing is limited by sampling tumor heterogeneity and fraught with tissue acquisition difficulties. A liquid biopsy-based PD-L1 assay could overcome these limitations of tissue PD-L1 testing.

**Methods:**

An observational cohort of patients with metastatic NSCLC treated with first-line ICI-based therapies were retrospectively assessed for a pre-planned endpoint of median and 3-year landmark overall survival (OS) based upon plasma cell-free RNA (cfRNA) *PD-L1* expression by a commercial exosome-free real-time qPCR assay or tissue PD-L1 protein expression (Dako 22C3) performed in CLIA/CAP accredited laboratories.

**Results:**

53 contemporaneous patients in 3 patient cohorts were compared with a median follow-up 34 months. 16 patients were cfRNA plasma *PD-L1* positive, including 6 (37 %) tissue PD-L1 negative or tissue quantity not sufficient for testing; 16 were plasma PD-L1 negative but tissue PD-L1 positive; 21 were both plasma and tissue PD-L1 negative. OS was identical whether positive plasma cfRNA *PD-L1* expression or positive tissue PD-L1 protein expression (median OS 15 months; 3-year landmark OS 30 %; hazard ratio (HR) 0.97; 95 % CI, 0.44–2.10; p-value = 0.93). Within the positive plasma PD-L1 cohort there was no differing OS whether tissue PD-L1 positive, negative, or unknown (median OS 15 months; 3-year landmark OS 30 %; HR 1.15; 95 % CI, 0.322–4.05; p-value = 0.81). Positive plasma cfRNA expression was associated with a numerically longer median and higher 3-year OS compared to patients lacking PD-L1 expression (median 15 months versus 8 months; 3-year landmark OS 30 % versus 15 %; HR 0.57, 95 % CI 0.26–1.20; p-value = 0.11).

**Conclusions:**

In this retrospective study of real-world metastatic NSCLC patients, plasma cfRNA *PD-L1* expression was similarly predictive of ICI benefit as tissue PD-L1 protein expression.

## 1 Introduction

Tissue programmed death ligand-1 (PD-L1) is the recognized immune biomarker of immune checkpoint inhibitor (ICI) treatment benefit in metastatic non-small cell lung cancer (NSCLC). However, tissue PD-L1 protein testing can be limited by tumor heterogeneity and fraught with tissue acquisition difficulties. Tissue testing is also limited to a one-time static assessment missing potential dynamic changes reflected in the evolving tumor biology of cancer. A plasma PD-L1 assay could overcome these tissue limitations and would also be able to assess dynamic changes of PD-L1 expression with treatment, recurrence, and upon progression guiding immune treatment decisions across the cancer treatment spectrum.

Prior plasma-based PD-L1 assays have not consistently been predictive of ICI treatment benefit. What is being tested by the plasma PD-L1 assay makes a difference. Plasma PD-L1 protein testing by enzyme-linked immunosorbent assays has not been predictive. Elevated levels of soluble protein PD-L1 were associated with poorer survival with ICI treatment in NSCLC and a meta-analysis of eight studies with over 1000 patients across a variety of solid tumors [1,2]. Secreted PD-L1 proteins have also been shown to contain decoy PD-L1 variants as a mediator of ICI treatment resistance [3]. Circulating tumor cell (CTC) based PD-L1 assays have shown an overall poor correlation with tissue PD-L1 expression and have also not been associated with a predictive ICI treatment benefit [[4], [5], [6]].

*PD-L1* gene amplification and mRNA expression are other potential PD-L1 testing options. Both have been associated with ICI treatment benefit. *PD-L1 (CD274)* copy number gains have been associated with significantly improved ICI treatment response rates of 67%–80 % and potential durable overall survival (OS) benefit. However, the very low frequency of *PD-L1* gene amplification of only 0.7–2.6 % greatly underestimates the potential benefit of ICI treatment limiting its clinical utility [7,8]. *PD-L1* polysomy is more frequent but was not predictive of ICI treatment benefit compared to patients without *PD-L1* polysomy copy number gain [8].

Tissue mRNA *PD-L1* expression in NSCLC is far more frequent than *PD-L1* gene amplification with 43–50 % rates of expression [9,10]. Conroy et al. concluded *PD-L1* mRNA expression is comparable to PD-L1 protein expression by immunohistochemistry (IHC) both analytically and clinically in predicting ICI response rates in NSCLC [11]. Fernandez et al. also reported a statistically significantly improved OS and long-term ICI benefit in chemo-immune treated metastatic NSCLC patients with high mRNA *PD-L1* expression compared to low expression. Conversely, low *PD-L1* mRNA expression had a high negative predictive value for absence of long-term ICI treatment benefit [12].

Although RNA is involved in protein synthesis, comparative studies have shown significant discordance between tissue mRNA *PD-L1* and protein PD-L1. There are a striking number of patients without identifiable PD-L1 protein expression yet mRNA *PD-L1* expression by PCR can be demonstrated. Venina et al. compared tissue *PD-L1* RNA by PCR in comparison to the Dako 22C3, Ventana SP263, and Ventana SP142 antibodies IHC staining in 167 NSCLC patients. When each antibody tumor proportion score (TPS) was <1 %, *PD-L1* mRNA still demonstrated expression in 50–56 % of patients. At the other end of the spectrum with TPS ≥50 %, higher *PD-L1* mRNA expression was demonstrated in 69–87 % of patients [13]. In another similar comparison to PD-L1 protein assays, Tsimafeyeu et al. found *PD-L1* mRNA expression in 43 % of 473 biobank tissue samples of NSCLC. Among those patients only half tested positive by these same three IHC antibodies with the authors concluding *PD-L1* mRNA expression has potential clinical utility in identifying PD-L1 when PD-L1 IHC protein is negative [10]. In both studies, lack of *PD-L1* mRNA expression demonstrated a high negative predictive value of 92–99 % with lack of PD-L1 protein expression. Tissue sampling heterogeneity missing PD-L1 protein IHC positive cells is the assumed explanation [14]. This suggests the utility of mRNA *PD-L1* expression as a potential better and certainly complementary predictive immune biomarker with PD-L1 protein. However, what potential advantages of tissue mRNA *PD-L1*, the limitations of any tissue testing remain.

Extracellular vesicle (EV) PD-L1 expression has been an effective blood-based immune biomarker. EV PD-L1 research assays have demonstrated that dynamic changes in EV PD-L1 were predictive of ICI treatment durability. Decreasing *PD-L1* mRNA expression by droplet digital PCR in plasma-derived exosomes was associated with an ICI response whereas an increase was seen in non-responders [15]. Another study identified the same response dynamics of EV PD-L1 expression and ICI treatment survival benefit but not in chemotherapy treated patients [16]. This emphasizes the potential utility of a plasma-based PD-L1 assay in assessing ICI treatment response, however neither EV PD-L1 assay was evaluated as a pre-treatment predictor of ICI benefit.

Plasma cell free mRNA (cfRNA) *PD-L1* expression by real-time polymerase chain reaction (RT-PCR) assays have been shown to be associated with ICI response. Ishiba et al. report cfRNA *PD-L1* by RT-PCR associated with ICI response in twelve patients [17]. Raez et al. reported both pre-treatment and dynamic changes of decreasing plasma cfRNA *PD-L1* by RT-PCR were associated with ICI treatment response rate in 52 metastatic NSCLC patients [18]. A retrospective patient experience demonstrated plasma cfRNA *PD-L1* by RT-PCR was associated with a statistically significant and clinically meaningful improved OS outcomes with ICI-based treatment compared to positive plasma cfRNA *PD-L1* patients treated with chemotherapy alone in metastatic NSCLC [19]. However, OS outcomes based upon plasma cfRNA *PD-L1* expression have not been fully characterized nor have outcomes of ICI treated patients been comparatively assessed with patients lacking plasma cfRNA *PD-L1* expression.

Our aim in this retrospective observational cohort study was to further evaluate the association of plasma cfRNA *PD-L1* expression by RT-PCR and first-line ICI-based OS clinical outcomes in metastatic NSCLC. We report the comparative median and a landmark 3-year OS of patient cohorts with positive plasma cfRNA *PD-L1* expression compared to positive tissue PD-L1 protein expression and to patients lacking PD-L1 expression.

## 2 Methods

This is a single-institution, retrospective observational study performed at the Brody School of Medicine at East Carolina University (Greenville, NC, USA) with patients treated at the Vidant Medical Center (now ECU Health Medical Center). Patients with pathologically confirmed metastatic NSCLC treated with first-line ICI-based treatment and with positive plasma cfRNA *PD-L1* expression and those with negative plasma cfRNA *PD-L1* expression with available tissue PD-L1 protein results with the Dako 22C3 monoclonal antibody were identified through the institutional thoracic oncology program database from November 2018 through July 2019 allowing sufficient follow-up to assess a landmark 3-year OS. Patients with stage I/II/III NSCLC, stage unknown, or with the presence of a targetable oncogenic driver mutations/fusions were excluded. There were no other clinical or laboratory exclusion criteria. No patients received definitive concurrent chemoradiation therapy or thoracic radiation therapy (RT). Palliative RT with either whole brain RT or Gamma Knife radiosurgery, or palliative stereotactic body RT were undertaken as indicated upon the recommendation of the treating oncologist. Patients were treated based upon the current available standard of care during that time period with the local treating oncologist making the final treatment decision. The study was approved by the Brody School of Medicine at East Carolina University Institutional Review Board (UMCIRB 20–002974-12 March 2021; UMCIRB 21–000046-30 July 2021).

Plasma for testing was collected at a single point in time before any treatment. Blood was collected in a single 10-ml ethylenediaminetetraacetic acid (EDTA) tube. Samples were shipped on ice in a commercial kit with plasma aliquots stored at −80° centigrade. The cfRNA *PD-L1* expression testing was performed at the Circulogene Clinical Laboratory Improvement Amendments (CLIA) certified/College of American Pathologists (CAP) accredited laboratory (Birmingham, AL – Pensacola, FL, USA). Circulogene is a commercial liquid biopsy vendor with a proprietary patented pre-analytical linear-in-situ-amplification technology. The cfRNA *PD-L1* Gene Expression assay is an exosome-free real-time PCR assay with four *PD-L1* PCR primers using beta-actin as a reference gene. The demonstrated limit of detection for cfRNA *PD-L1* was 1.0 copy/uL.

OS was assessed from the date of diagnosis and either death or censored follow-up with a data cut-off of March 9, 2022. Median follow-up was 34 months. OS analysis was performed by AnalystSoft StatPlus Kaplan-Meier and hazard ratio (HR) survival analysis. The pre-specified endpoint was median and 3-year OS.

## 3 Results

53 metastatic NSCLC patients treated with first-line ICI-based treatment fulfilled the inclusion criteria. Patients were comparatively assessed within three cohorts: (i) Plasma cfRNA *PD-L1* positive patients; (ii) tissue PD-L1 protein positive patients; (iii) PD-L1 negative patients by both plasma cfRNA and tissue protein.

The (i) ‘plasma PD-L1 positive’ cohort consisted of sixteen patients who demonstrated plasma cfRNA *PD-L1* expression and were treated with first-line ICI-based therapies. Thirteen patients received combination anti-PD-1/L1 ICI plus chemotherapy regimens and three patients anti-PD-1/-L1 ICI alone. No patients received anti-CTLA-4 agents. Tissue PD-L1 TPS was reported as ≥ 50 % in six patients and ≥1 % in four patients. Six of the total IO cohort of sixteen patients (37 %) were either tissue PD-L1 negative or unknown due to tissue quantity not sufficient (QNS) for testing. The (ii) ‘tissue PD-L1 positive’ cohort consisted of sixteen contemporaneously identified patients receiving first-line ICI treatment who were tissue PD-L1 protein positive. Eleven patients received combination anti-PD-1/L1 plus chemotherapy. Five received anti-PD-1/L1 alone. No patients received anti-CTLA-4 agents. PD-L1 TPS was 1–49 % in six and ≥50 % in ten, with five of those ten ≥90 %. All of these patients were plasma PD-L1 negative. The (iii) ‘PD-L1 negative’ cohort consisted of 21 contemporaneously identified patients receiving first-line ICI treatment who were PD-L1 negative by both plasma cfRNA and tissue protein. All patients received anti-PD-1/L1 plus chemotherapy regimens. No patients received anti-CTLA-4 agents. Table 1 summarizes the three patient cohorts.

**Table 1:** Clinical summary of each patient cohort.

	Plasma PD-L1 Positive	Tissue PD-L1 Positive	PD-L1 Negative
	N = 16	N = 16	N = 21
Gender	Female = 8 Male = 8	Female = 7 Male = 9	Female = 10 Male = 11
Age	Median 65 (range 54–85)	Median 68 (range 50–90)	Median 66 (range 58–85)
Histology	Non-squamous = 12 Squamous = 4	Non-squamous = 12 Squamous = 4	Non-squamous = 16 Squamous = 5
ECOG PS	PS 1 = 7 PS ≥ 2 = 9	PS 0/1 = 10 PS ≥ 2 = 6	PS 1 = 13 PS ≥ 2 = 8
treatment	Chemo-ICI = 13 ICI alone = 3	Chemo-ICI = 11 ICI alone = 5	Chemo-ICI = 21
Tissue PD-L1 TPS	≥50 % = 6 1–49 % = 4 <1 % = 3 QNS = 3	≥90 % = 5 ≥50 % = 10 1–49 % = 6	Negative = 21

**Fig. 1:**
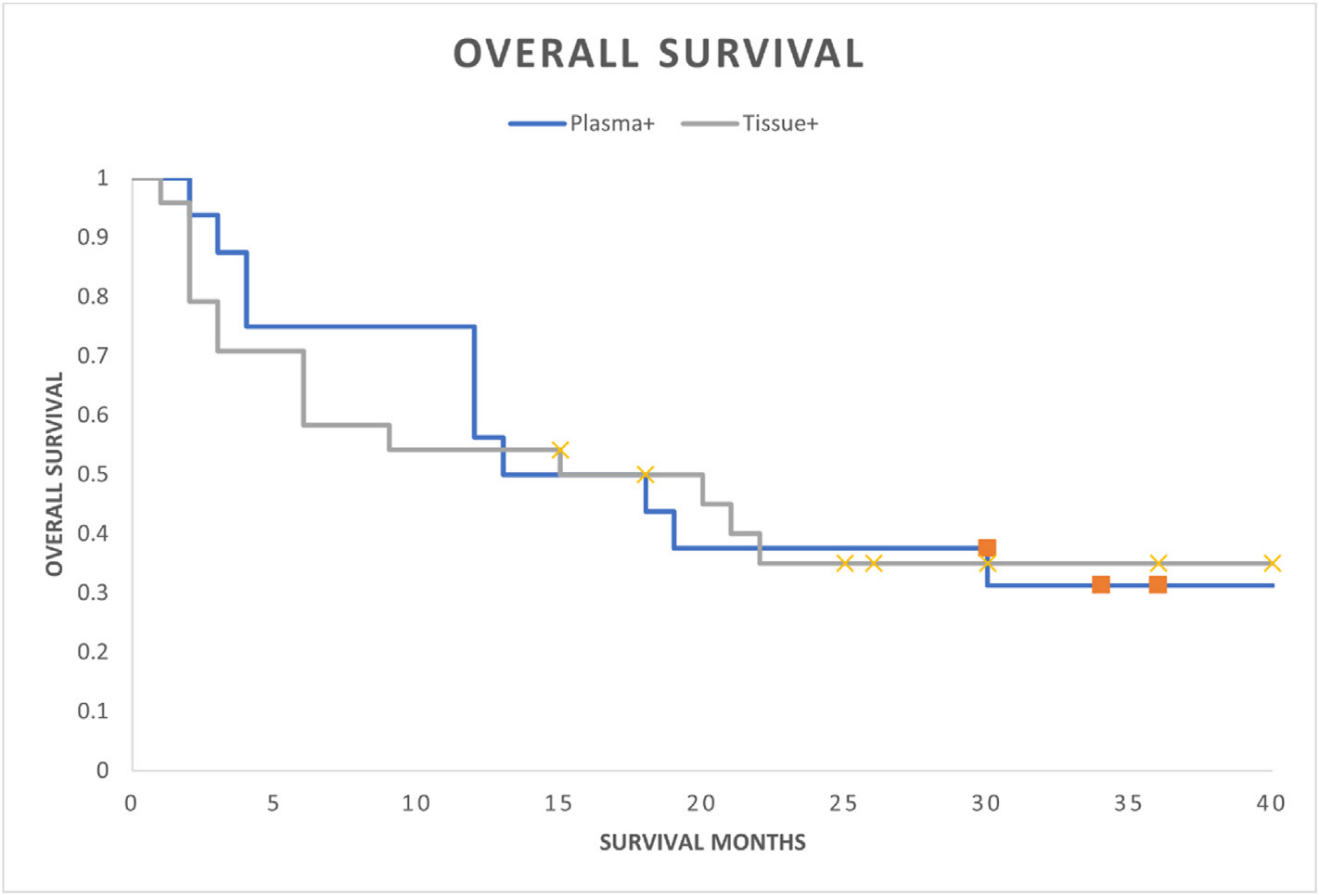
Overall survival comparison between positive plasma cfRNA *PD-L1* patients compared to positive tissue PD-L1 protein patients.

**Fig. 2:**
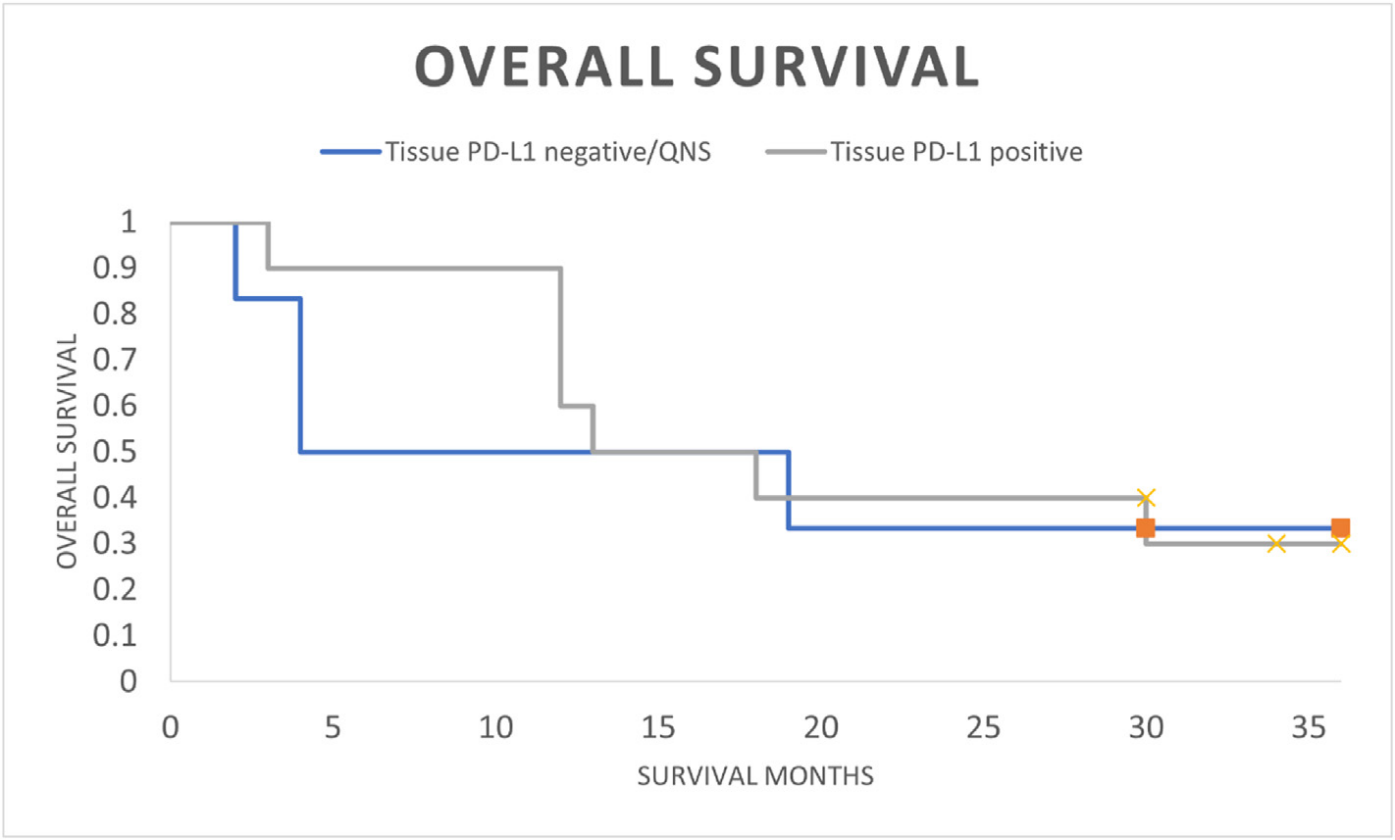
Overall survival of positive plasma cfRNA *PD-L1* patients whether tissue PD-L1 positive or tissue PD-L1 negative/unknown.

OS outcomes were identical in the plasma cfRNA *PD-L1* positive and tissue PD-L1 protein positive patient cohorts (median OS 15 months; landmark 3-year OS 30 %; HR 0.97; 95 % CI, 0.44–2.10; p-value = 0.93) (Fig. 1). Within the plasma PD-L1 positive cohort there were no differing OS outcomes whether tissue PD-L1 positive, negative, or unknown (median OS 13–16 months; 3-year landmark OS 30 %; HR 1.15; 95 % CI, 0.32–4.05) (Fig. 2).

**Fig. 3:**
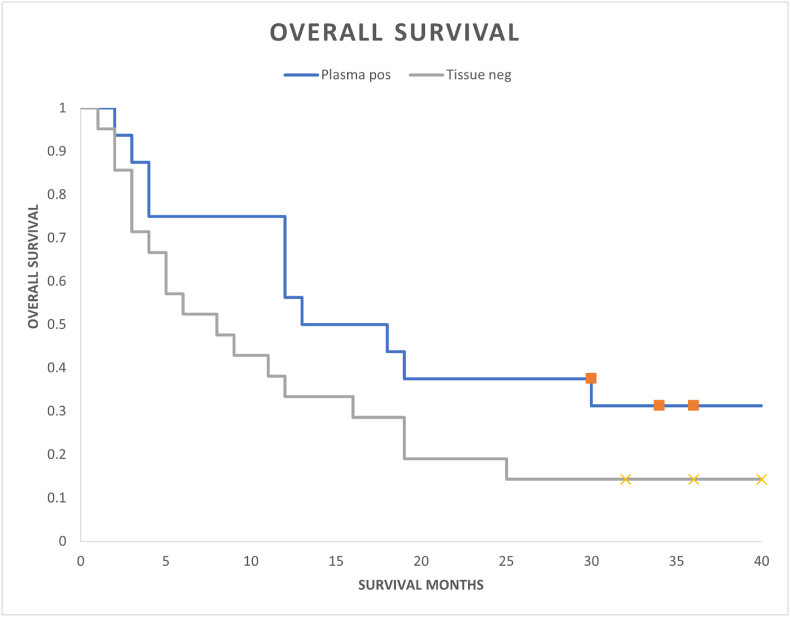
Overall survival of positive plasma cfRNA *PD-L1* patients compared to patients lacking plasma/tissue PD-L1 expression.

Positive plasma PD-L1 patients demonstrated numerically longer median and higher 3-year OS compared with patients lacking PD-L1 expression (median OS 15 months versus 8 months; landmark 3-year OS 30 % versus 15 %; HR 0.56; 95 % CI, 0.27–1.17; p-value = 0.11) (Fig. 3). Although not statistically significant, the OS curves separated early with retained OS separation throughout the follow-up period suggesting the modest patient numbers in each cohort limiting statistical significance.

## 4 Discussion

Improving predictive immune biomarker assays is clinically important to better predict ICI treatment benefit and extend that benefit to more patients. A plasma PD-L1 assay would not be limited by sampling heterogeneity or require tissue acquisition. It would also allow dynamic monitoring of ICI response and easily reassess PD-L1 expression upon cancer recurrence and/or progression. The International Association for the Study of Lung Cancer (IASLC) supports complementary tissue and plasma molecular testing for a full molecular assessment of driver mutations and fusions. The IASLC consensus statement went further advocating a ‘plasma first’ approach for this molecular testing [20]. A similar complementary testing paradigm of PD-L1 testing could be undertaken with a predictive plasma PD-L1 assay.

A previous real-world patient experience in advanced NSCLC demonstrated a significantly improved median and landmark 3-year OS with ICI-based treatment compared to chemotherapy alone in patients with positive plasma cfRNA *PD-L1* expression [19]. Those clinical outcomes mirror the same predictive ICI treatment benefit with tissue PD-L1 compared to chemotherapy in seminal ICI clinical trials [21,22]. This expanded patient cohort experience of first-line ICI treated metastatic NSCLC patients further demonstrates that plasma cfRNA *PD-L1* expression by RT-PCR is associated with favorable ICI treatment outcomes. The OS was identical whether patients were positive with plasma cfRNA *PD-L1* or tissue PD-L1 protein. Both plasma and tissue PD-L1 assays were associated with a 30 % landmark 3-year OS. When positive plasma cfRNA *PD-L1* expression was present, the OS benefit of ICI treatment was the same, whether tissue PD-L1 protein was positive, negative, or tissue QNS for testing. Positive plasma cfRNA *PD-L1* patients also achieved an early and sustained separation of OS benefit resulting in a numerically improved median and landmark 3-year OS survival benefit with ICI treatment compared to patients who lacked PD-L1 expression by both plasma cfRNA and tissue protein assays.

Predictive immune biomarkers remain important to identify patients who will benefit from ICI-based treatment [23]. These biomarkers need to continue to evolve and improve. A plasma PD-L1 assay would be a potential step in that improvement. Complementary tissue and plasma testing of genomic tumor biology testing is supported by multiple studies in NSCLC, as tissue testing only approaches will miss a significant number of alterations [[24], [25], [26]]. That appears to also be true for PD-L1. Tissue PD-L1 was either negative or unknown when plasma PD-L1 was positive in 37 % of this patient population. Tissue *PD-L1* mRNA has been reported to be expressed in up to half of patients even when tissue PD-L1 protein TPS is negative. With tissue PD-L1 protein testing alone, a significant number of patients with *PD-L1* upregulation will be missed. This real-world data provides a framework of the potential clinical utility of plasma cfRNA *PD-L1* expression. Complementary tissue and plasma PD-L1 testing would have clinical impact, especially when tissue PD-L1 protein is negative or QNS for testing.

This study has well acknowledged limitations. It only provides retrospective single-institutional observational outcomes data in a modest number of patients. The outcome results are reported solely based upon PD-L1 expression not assessing other potential confounding clinical or genomic OS variables. Most patients in both the plasma positive and tissue positive PD-L1 patient cohorts were treated with combined ICI with chemotherapy and not ICI monotherapy. A biomarker comparison of the plasma cfRNA *PD-L1* by Ct-value PCR expression correlation with tissue PD-L1 TPS or ICI treatment outcomes is clinically important but was not able to be assessed in this retrospective study. Prospective research is needed to further assess any immune tumor biology and ICI treatment benefit differences of plasma *PD-L1* RNA and tissue PD-L1 protein levels of expression to provide insight into the full potential clinical utility of plasma PD-L1 testing.

A strength of this data is that all three patient cohorts represent a real-world patient experience including ECOG performance status of 2 or greater with consistent PD-L1 testing and ICI treatment. There is now an evolving understanding that imaging-based response rates and progression free survival (PFS) are not consistent surrogates of true ICI treatment OS benefit. A pooled analysis of first-line ICI randomized trials failed to show a strong correlation between PFS or response rates with OS. Mature OS data is the gold standard endpoint for first-line ICI trials [27,28]. Given this, it was felt that only ICI treatment OS outcomes would reflect the potential predictive immune biomarker benefit of plasma cfRNA *PD-L1* expression. This study reflects mature OS data with prolonged follow-up and adds to the published experience of plasma cfRNA *PD-L1* expression and ICI treatment outcomes.

## 5 Conclusion

In a real-world population of symptomatic metastatic NSCLC patients, positive plasma cfRNA *PD-L1* expression was associated with favorable outcome findings with first-line ICI treatment. Either tissue PD-L1 protein expression by IHC or plasma cfRNA *PD-L1* expression by RT-PCR was associated with an identical OS ICI treatment benefit. Patients with positive plasma cfRNA *PD-L1* expression were also associated with better ICI treated OS compared to patients with a lack of cfRNA *PD-L1* expression. This data lends support for needed prospective validation and expanded study of the potential clinical utility and benefit of plasma cfRNA *PD-L1* as a predictive immune biomarker.

## Funding

This research received no external funding.

## Author contributions

Paul Walker: Conceptualization, Methodology, Software, Formal Analysis, Resources, Writing- Original Draft, Writing-Review and Editing, Visualization, Sriraksha Jayananada: Validation, Data Curation, Writing-Review and Editing, Melisa Pasli:Validation, Data Curation, Writing-Review and Editing, Mahvish Muzaffar: Writing-Review and Editing, Supervision, Project Administration.

## Declaration of competing interest

PW is Emeritus faculty at the Brody School of Medicine at East Carolina University and a current employee of Circulogene. The other authors have no conflicts of interest to declare.
